# Genetic Association of CHAT rs3810950 and rs2177369 Polymorphisms with the Risk of Alzheimer's Disease: A Meta-Analysis

**DOI:** 10.1155/2016/9418163

**Published:** 2016-08-15

**Authors:** Yong Liu, Qicong Chen, Xu Liu, Mengmeng Dou, Silu Li, Jiahui Zhou, Hong Liu, Yongfu Wu, Zunnan Huang

**Affiliations:** ^1^Dongguan Scientific Research Center, Guangdong Medical University, Dongguan, Guangdong 523808, China; ^2^School of Pharmacy, Guangdong Medical University, Dongguan, Guangdong 523808, China; ^3^Key Laboratory for Research and Development of Natural Drugs of Guangdong Province, Zhanjiang, Guangdong 524023, China; ^4^Department of Biochemistry and Molecular Biology, Guangxi Medical University, Nanning, Guangxi 530021, China; ^5^The Second School of Clinical Medicine, Guangdong Medical University, Dongguan 523808, China; ^6^Key Laboratory for Medical Molecular Diagnostics of Guangdong Province, Dongguan, Guangdong 523808, China; ^7^School of Basic Medicine, Guangdong Medical University, Dongguan, Guangdong 523808, China; ^8^Affiliated Hospital of Guangdong Medical University, Zhanjiang, Guangdong 524023, China

## Abstract

Choline acetyltransferase (CHAT) rs3810950 and rs2177369 polymorphisms have been implicated in susceptibility to Alzheimer's disease (AD). Due to the inconsistent results from previous studies, a meta-analysis was performed to estimate the association between these polymorphisms and AD risk more precisely. Pooled results of our meta-analysis indicated CHAT rs2177369 polymorphism was correlated with decreasing AD risk in one of five genetic models (dominant: OR = 0.77, 95% CI: 0.62–0.96), while rs3810950 mutant was associated with AD development in three models (allelic: OR = 1.18, 95% CI: 1.01–1.37, homozygous: OR = 1.63, 95% CI: 1.09–2.42, and recessive: OR = 1.65, 95% CI: 1.20–2.26). In subgroup analysis by ethnicity, the association between CHAT rs3810950 polymorphism and AD risk was just found in the recessive model (OR = 1.47, 95% CI: 1.05–2.07) among Caucasians, while four genetic models (allelic: OR = 1.23, 95% CI: 1.01–1.48; homozygous: OR = 2.24, 95% CI: 1.48–3.39; dominant: OR = 1.21, 95% CI: 1.06–1.40; and recessive: OR = 2.18, 95% CI: 1.45–3.29) assumed this association in Asians. In conclusion, our meta-analysis indicated CHAT rs2177369 polymorphism might play a protective role in AD, while rs3810950 variant was a risk factor for AD but its single heterozygous mutations might not influence susceptibility to AD.

## 1. Introduction

Alzheimer's disease (AD) is a neurodegenerative disorder characterized by severe damage of cognition. It is the most common form of age-related dementia [1]. Today, 35 million patients fight against dementia and most of them suffer from AD [1, 2]. This brings huge losses to the social economy and seriously affects the long-term health-care system. Neuropathology of AD is characterized by the accumulation of extracellular *β*-amyloids (A*β*) in plaques and intracellular hyperphosphorylated tau protein [3]. Although the causes for the development of AD are still unclear, many studies showed that AD was triggered by multiple genetic and environmental factors [2, 4–6].

Genetic studies revealed that genetic factors played significant roles in the development of Alzheimer's disease [5]. Recently, several studies, including the large-scale genome-wide association studies (GWAS) of AD, have reported some susceptibility genes such as Apolipoprotein E (ApoE), Bridging Integrator 1 (BIN1), Clusterin (CLU), complement component receptor 1 (CR1), and choline O-acetyltransferase (CHAT) [7–10]. Among them, CHAT is the key enzyme responsible for the synthesis of a neurotransmitter acetylcholine and the target for many effective pharmacological therapies of AD [11].

CHAT gene has several genetic polymorphic loci such as rs1880676, rs2177369, rs868750, and rs3810950. In this paper, we focus only on the influence of the rs3810950 (G>A) and rs2177369 (A>G) polymorphisms on AD risk. We do not include other CHAT polymorphisms for this meta-analysis because all the other available polymorphisms do not meet the performance standard of conducting a mate-analysis due to their limited case-control studies.

The mutation of A/G in both rs3810950 and rs2177369 is fascinating due to its functionality. These polymorphisms in the CHAT gene may affect the synthesis of the enzyme, thereby amplifying the cholinergic neurotransmission deficits in AD [12]. The rs3810950 polymorphism has been proven to be associated with Alzheimer's disease in nine studies [10, 12–19]. In these studies, the gene variation was found to associate with Alzheimer's disease among people in Asia, America, and five European countries. However, two other studies identified no association between CHAT rs3810950 and AD in British people [20, 21]. Similarly, three previous articles, which investigated the relationship between rs2177369 and AD risk in the British and Italian population, respectively, also provided inconsistent results [21–23]. These controversial results from the earlier reports of different geographic areas might be caused by the relatively small size of each individual study and its low power to detect the true effect. We thus conducted a meta-analysis to give a more precise estimation of the association between these two CHAT polymorphisms and AD susceptibility.

## 2. Methods and Materials

### 2.1. Literature Search and Inclusion Criteria

Two investigators searched the PubMed, Embase, CNKI, Wanfang, and AlzGene databases to find all relevant records using the following keywords: “Choline acetyltransferase OR CHAT”, “Alzheimer's disease OR AD” and “polymorphism OR polymorphisms OR variant OR mutation”. The searches were last updated on May 15, 2016. Two authors took responsibility for literature searches to ensure the integrity of the data collection.

The inclusion criteria to select eligible articles in this meta-analysis were as follows: (1) the association of CHAT rs3810950 or rs2177369 polymorphism with Alzheimer's disease; (2) a case-control design; (3) complete genotype data including the number of homozygous mutant, heterozygous and wild genotypes to calculate ORs. On the other hand, the literatures were excluded if they met any of the following criteria: (1) abstracts, editorials, review articles, and unrelated meta-analyses; (2) studies without polymorphisms; (3) studies with other diseases or other polymorphisms; (4) studies with incomplete genotype data. Only one could be accepted if the publications were duplicated. Any disagreement regarding the inclusion of articles was resolved by discussion among the authors.

### 2.2. Data Extraction

One author extracted the following information from each study: (1) the first author's name; (2) the year of publication; (3) the country and ethnicity of the participants (patients and controls); (4) the number of Alzheimer's disease cases and controls; (5) the frequency of genotypes in AD cases and controls; (6) genotyping method; (7) diagnosis criteria of AD. Then, another author checked the data carefully to ensure they are complete and correct. In case-control studies, Hardy Weinberg Equilibrium (HWE) was used for quality assessment of genotype data. A high-quality study was considered that its control group conformed to HWE. A study without HWE in controls was defined as a low quality one. Low quality studies were excluded in the sensitivity analysis. Newcastle-Ottawa Scale (NOS) criteria [24] were used to evaluate the quality of the case-control studies included in the meta-analysis. The evaluation of content in the NOS was classified into three independent aspects: object selection, comparability, and exposure assessment. In this retrospective analysis, an included study should get at least five points [25] in the NOS quality assessment.

### 2.3. Statistical Analysis

In this meta-analysis, the pooled odds ratios (ORs) with 95% confidence intervals (CIs) were used to estimate the association of CHAT rs3810950 or rs2177369 polymorphisms with the risk of AD. We did not use environmental factors to adjust the poor ORs due to the very limited information provided in each of the included studies. We performed this meta-analysis using five genetic models: the allelic, homozygous, heterozygous, dominant, and recessive model. Chi-square test and *I* ^2^ test were used to calculate the heterogeneity of these genetic models. A *P* value <0.05 and/or *I* ^2^ > 50% indicated substantial heterogeneity, and then a random effect model was used; otherwise, a fixed effect model was used to calculate the ORs and 95% CIs of any genetic model with unobserved heterogeneity [26, 27]. Additionally, meta-regression based on the covariates of sample size, ethnicity, and genotyping method was adopted to explore the source of heterogeneity. Subgroup analysis was also conducted according to ethnicity and genotyping method. At the same time, we did a stratified analysis by ApoE-*ε*4 status.

Statistical power analysis was executed to estimate the suitability of the sample size employed to the power of the study. We assumed an unmatched case-control design and considered a two-sided *P* value of 0.05. “Venice criteria” [28] (Table S1, see Supplementary Material available online at http://dx.doi.org/10.1155/2016/9418163) were also applied to assess the credibility of the cumulative evidence of each meta-analyzed association under the genetic models we investigated. The evidence level is graded as strong, moderate, or weak.

Sensitivity analysis was applied to investigate the influence of the individual studies to the pooled results by omitting one study at a time. Both Begg's and Egger's tests were used to assess publication bias and a *P* value of less than 0.01 was considered statistically significant. The trim and fill method was also employed to identify and correct funnel plot asymmetry arising from publication bias. Data analysis was performed by professional software Review Manager 5.3 (Cochrane Informatics and Knowledge Management Department), STATA 14.0 (Stata Corporation College Station, Texas, USA), and Quanto software package (Version 1.2.4, http://biostats.usc.edu/software).

## 3. Results

### 3.1. Characteristics of the Studies

**Figure 1:**
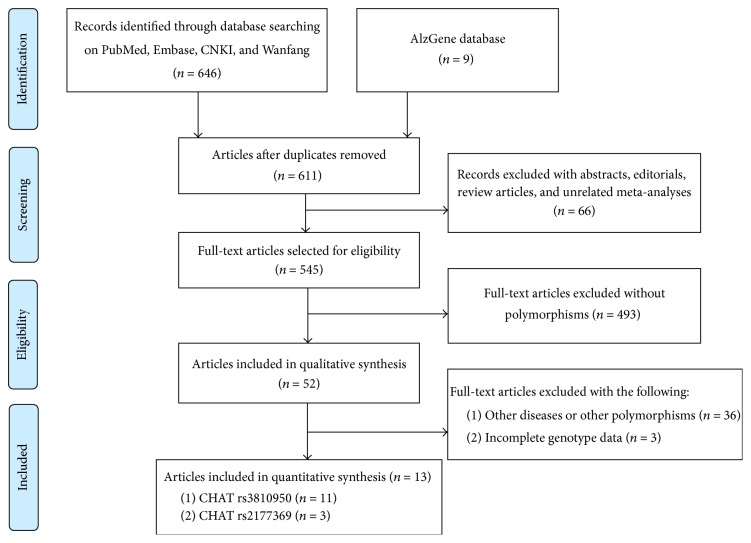
Flow diagram of the process used to select eligible studies.

As shown in Figure 1, 655 related articles were first discovered from database searching up to May 15, 2016. Then, 611 articles were obtained after the duplicated publications were weeded out. Among them, 66 were abstracts, editorials, review articles, or unrelated meta-analyses, 493 lacked polymorphisms, and 39 related to other diseases and polymorphisms or had incomplete genotype data. Thus, we further discarded these studies (598) according to the exclusion criterions. Finally, we got thirteen eligible articles for our meta-analysis, which included 11 studies related to CHAT rs3810950 polymorphism and four studies linked to CHAT rs2177369 polymorphism. The baseline characteristics of all of these studies are listed in Table 1. The included studies conformed to HWE except for two: one reported by Mubumbila et al. [13] on CHAT rs3810950 polymorphism and the other one as Cook (1) study by Cook et al. [21] on CHAT rs2177369 polymorphism. Statistical power based on the given sample size of each study ranged from 5.00% to 90.29% under the dominant model and from 5.73% to 98.68% under the recessive model. The NOS results showed that the quality score of all the included studies satisfied the standard to reach five points or more (Table 2).

### 3.2. A Meta-Analysis of CHAT rs3810950 Polymorphism with AD Risk

**Figure 2:**
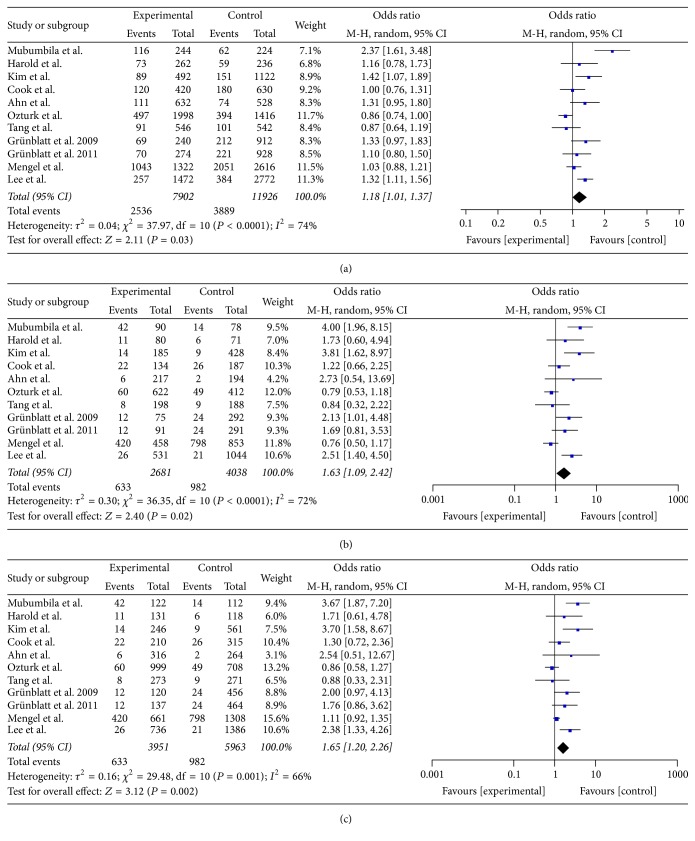
Forest plots of CHAT rs3810950 polymorphism and AD risk in three genetic models. (a) The allelic model (A versus G); (b) the homozygous model (AA versus GG); (c) the recessive model (AA versus AG + GG).

In this meta-analysis, a total of 11 studies [10, 12–21] involving 3951 patients and 5963 controls were included to investigate the associations between CHAT rs3810950 and the risk of Alzheimer's disease (as shown in Table 1). Combined data showed that CHAT rs3810950 polymorphism was associated with an increased risk of AD in three of five genetic models (allelic A versus G: OR = 1.18, 95% CI: 1.01–1.37, *P* = 0.03; homozygous AA versus GG: OR = 1.63, 95% CI: 1.09–2.42, *P* = 0.02; and recessive AA versus AG + GG: OR = 1.65, 95% CI: 1.20–2.26, *P* < 0.01) (Figure 2) but no association was observed in the remaining two models (heterozygous AG versus GG: OR = 0.99, 95% CI: 0.90–1.10, *P* = 0.87; dominant AA + AG versus GG, OR = 1.08, 95% CI: 0.92–1.28, *P* = 0.34) (Table 3). Power analysis on the pooled sample size showed that the statistical power was 46.23% and 99.99%, respectively, in the dominant and recessive models. In general, our meta-analysis demonstrated that CHAT rs3810950 polymorphism increased the risk of Alzheimer's disease but only a heterozygous mutation of rs3810950 might not influence the susceptibility to AD.

### 3.3. Subgroup Analysis

The results from a subgroup analysis by ethnicity in Caucasians and Asians for all five genetic models are shown in Table 3. Only the recessive model (AA versus GG + GA: OR = 1.47, 95% CI: 1.05–2.07, *P* = 0.03) established the association of CHAT rs3810950 polymorphism with AD risk among Caucasians, while four genetic models (allelic A versus G: OR = 1.23, 95% CI: 1.01–1.48, *P* = 0.04; homozygous AA versus GG: OR = 2.24, 95% CI: 1.48–3.39, *P* < 0.01; dominant AA + AG versus GG, OR = 1.21, 95% CI: 1.06–1.40, *P* < 0.01; and recessive AA versus GG + GA: OR = 2.18, 95% CI: 1.45–3.29, *P* < 0.01) assumed this association among Asians. The statistical power of an Asian subgroup was 77.90% and 99.45% and that of a Caucasian subgroup was 6.34% and 99.99% calculated from power analysis, respectively, under the dominant and recessive models. Thus, only the homozygous mutant genotype AA might be related with the susceptibility to AD in a Caucasian population but only the heterozygous genotype AG might not be linked to increasing AD risk in an Asian population. In addition, a further analysis of studies involving British only indicated that no association between CHAT rs3810950 polymorphism and Alzheimer's disease was found in British people for all five genetic models (Table S2 in Supporting Information). The statistical power calculated for the British group was 5.41% and 26.56% under the dominant and recessive models, respectively.

In the subgroup analysis according to the genotyping method [29], conflict results were obtained based on two different subgroups known as quantitative PCR and nonquantitative PCR (Table 3). The pooled ORs calculated for the quantitative PCR group showed that CHAT rs3810950 polymorphism contributed to increasing AD risk in three of five genetic models (allelic A versus G: OR = 1.32, 95% CI: 1.05–1.65, *P* = 0.02; homozygous AA versus GG: OR = 1.89, 95% CI: 1.00–3.55, *P* = 0.05; and recessive AA versus GG + GA: OR = 1.94, 95% CI: 1.18–3.19, *P* = 0.01) while no association was found in all the five genetic models for the nonquantitative PCR group (Table 3). The statistical power of a quantitative PCR subgroup was 77.24% and 99.99% and that of a nonquantitative PCR subgroup was 5.11% and 75.03%, respectively, under the dominant and recessive models.

### 3.4. Stratified Analysis

Because four included studies in this meta-analysis provided the genotype data of CHAT rs3810950 polymorphism and ApoE-*ε*4 allele [10, 12, 14, 15], we further operated a risk-stratification analysis to calculate the association of rs3810950 polymorphism with AD based on the absence and presence of ApoE-*ε*4. The combined influence of CHAT rs3810950 polymorphism and ApoE-*ε*4 allele on Alzheimer's disease is shown in Table 4. For all the comparisons, the GG + GA genotype within non-ApoE-*ε*4 carriers served as a reference. Among ApoE-*ε*4 carriers, individuals with the GG + GA genotype showed a significantly increased risk of Alzheimer's disease (OR = 3.46, 95% CI: 1.78–6.71, *P* < 0.001). A substantial interaction was also found between ApoE-*ε*4 carriers and the AA genotype (OR = 4.87, 95% CI: 1.67–14.22, *P* = 0.004). All of these evidences manifested that ApoE-*ε*4 allele could be a vital factor in the Alzheimer's disease caused by CHAT rs3810950 polymorphism. Under the existence of ApoE-*ε*4, the risk of Alzheimer's disease increased notably when the genotype of rs3810950 was GG + GA or AA.

### 3.5. Strength of the Evidence

When Venice criteria were applied to assess credibility, results under all five genetic models were graded as “A” for “amount of evidence” of all meta-analyses except “B” for British subgroup analysis, “A,” “B,” or “C” for “replication consistency,” and “A” for “protection from bias” of the overall analysis (Table S3). These results suggested that there was moderate or weak evidence of the association between rs3810950 polymorphism and AD susceptibility.

### 3.6. A Meta-Analysis between CHAT rs2177369 Polymorphism and AD Risk

**Figure 3:**
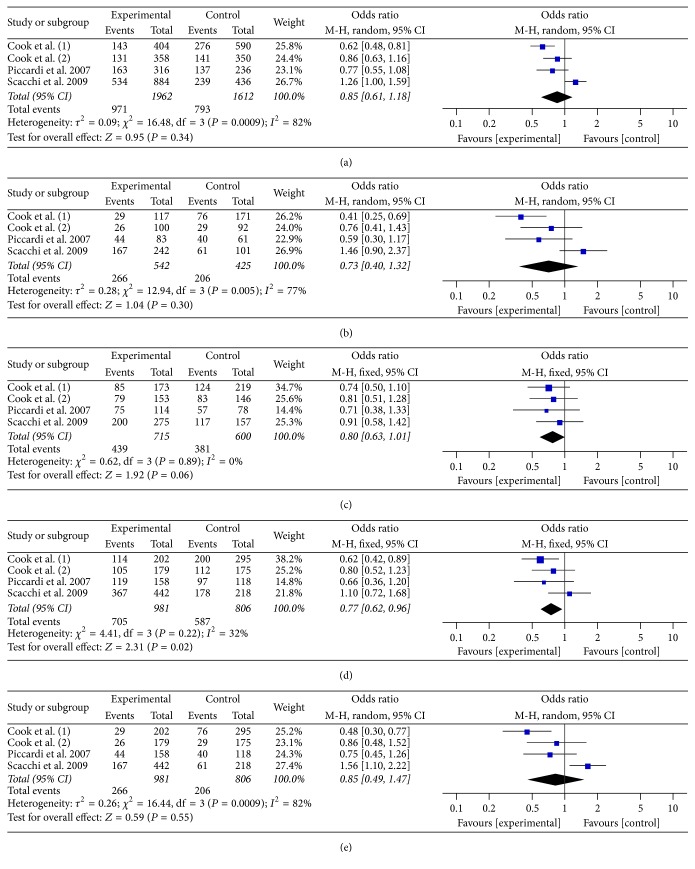
Forest plots of CHAT rs2177369 polymorphism and AD risk in five genetic models. (a) The allelic model (G versus A); (b) the homozygous model (GG versus AA); (c) the heterozygous model (GA versus AA); (d) the dominant model (GG + GA versus AA); (e) the recessive model (GG versus GA + AA).

A meta-analysis of the association between CHAT rs2177369 polymorphism and AD risk included four independent studies [21–23] with a total of 981 cases and 806 controls (as shown in Table 1). Combined data revealed that CHAT rs2177369 polymorphism was correlated with a decreased risk of AD in the dominant model (GG + GA versus AA: OR = 0.77, 95% CI: 0.62–0.96, *P* = 0.02, statistical power = 69.05%) but no connection was detected in the rest four genetic models (allelic G versus A: OR = 0.85, 95% CI: 0.61–1.18, *P* = 0.34; homozygous GG versus AA: OR = 0.73, 95% CI: 0.40–1.32, *P* = 0.30; heterozygous GA versus AA: OR = 0.80, 95% CI: 0.63–1.01, *P* = 0.06; recessive GG versus GA + AA: OR = 0.85, 95% CI: 0.49–1.47, *P* = 0.55, statistical power = 30.29%) (Table 5, Figure 3). Thus, the overall analysis indicated that CHAT rs2177369 polymorphism could reduce the risk of AD but the association might be only slightly correlated.

We also performed two subanalyses which excluded Cook (1) and Scacchi studies, respectively, because these two studies provided entirely opposite effects of CHAT rs2177369 polymorphism on AD risk [21, 23]. The meta-analysis from the exclusion of Cook (1) study showed that CHAT rs2177369 mutant was not associated with AD risk in all five genetic models (allelic G versus A: OR = 0.96, 95% CI: 0.70–1.31, *P* = 0.78; homozygous GG versus AA: OR = 0.91, 95% CI: 0.52–1.59, *P* = 0.73; heterozygous GA versus AA: OR = 0.83, 95% CI: 0.62–1.10, *P* = 0.19; dominant GG + GA versus AA: OR = 0.87, 95% CI: 0.67–1.14, *P* = 0.32, statistical power = 19.62%; recessive GG versus GA + AA: OR = 1.04, 95% CI: 0.63–1.71, *P* = 0.88, statistical power = 5.97%) (Table 5, Figure S1). On the other hand, the meta-analysis from the exclusion of Scacchi study showed that CHAT rs2177369 variant was statistically significant associated with decreasing AD risk in all five genetic models (allelic G versus A: OR = 0.73, 95% CI: 0.61–0.86, *P* < 0.01; homozygous GG versus AA: OR = 0.54, 95% CI: 0.39–0.76, *P* < 0.01; heterozygous GA versus AA: OR = 0.76, 95% CI: 0.58–0.99, *P* = 0.05; dominant GG + GA versus AA: OR = 0.68, 95% CI: 0.53–0.88, *P* < 0.01, statistical power = 86.83%; recessive GG versus GA + AA: OR = 0.65, 95% CI: 0.48–0.87, *P* < 0.01, statistical power = 73.79%) (Table 5, Figure S2). Thus, the sub-analysis of the exclusion of Cook (1) study showed that CHAT rs2177369 polymorphism did not affect AD risk while that from the exclusion of Scacchi study indicated that this polymorphism played a protective role in the Alzheimer's disease.

### 3.7. Heterogeneity, Meta-Regression, Sensitivity Analysis, and Publication Bias

Our meta-analysis showed evidence of genetic heterogeneity in all the genetic models of the two polymorphisms except for CHAT rs2177369 polymorphism in the heterozygous model (*I* ^2^ = 0%, as shown in Tables 3 and 5). Through the calculation of between-study heterogeneity in clinical AD samples, we found significant heterogeneity among studies on CHAT rs3810950 polymorphism in the allelic model (*I* ^2^ = 74%), homozygous model (*I* ^2^ = 72%), dominant model (*I* ^2^ = 62%), and recessive model (*I* ^2^ = 66%) and on CHAT rs2177369 polymorphism in the allelic model (*I* ^2^ = 82%), homozygous model (*I* ^2^ = 77%), and recessive model (*I* ^2^ = 82%). Meanwhile, moderate heterogeneity was observed in the remaining two models as the heterozygous model (*I* ^2^ = 37%) on CHAT rs3810950 polymorphism and the dominant model (*I* ^2^ = 32%) on CHAT rs2177369 polymorphism. On CHAT rs3810950 polymorphism, the results of the meta-regression showed that the sample size, ethnicity, and genotyping method did not contribute to the heterogeneity of genetic models (data not shown here). Sensitivity analysis was then performed to assess the impact of the independent studies which caused obvious heterogeneity in those four models on CHAT rs3810950 polymorphism. We explored the influence of these studies on the pooled OR by removing each one at a time and found no significant change of our meta-analysis results. We did not perform both meta-regression and sensitivity analysis on CHAT rs2177369 polymorphism due to only four case-control studies involved.

We further performed Begg's and Egger's tests to assess publication bias in the study of the genetic association between CHAT rs3810950 polymorphism and AD risk. As shown in Table S4, no obvious publication bias was detected according to the obtained *P* values for these genetic models. In addition, we did not observe any obvious asymmetry from the shape of Begg's funnel plot (Figure S3). In general, the effect of publication bias could be negligible in the included studies on CHAT rs3810950 polymorphism. However, we estimated the risk of publication bias neither in the subgroup analyses nor in the meta-analysis on CHAT rs2177369 polymorphism because the number of case-control studies in these studies was less than ten.

## 4. Discussion

The association of CHAT rs3810950 polymorphism with Alzheimer's disease was previously reported in one published meta-analysis [30]. However, the earlier work only included three case-control studies with 1183 incident AD patients and 1705 controls. In order to give a more precise estimation of the association between CHAT rs3810950 polymorphism and AD, we collected 11 eligible studies with a total of 3951 cases and 5963 controls to perform this meta-analysis. Based on the enriched data we got, five genetic models were carried out to further clarify the impact of rs3810950 variant on AD. As a result, our meta-analysis showed that CHAT rs3810950 polymorphism was associated with the risk of Alzheimer's disease but such an association might not exist for only single-allele mutant in the general population (Figure 2 and Table 3). In other words, people with the homozygous mutant genotype (AA) should have a much higher risk of developing Alzheimer's disease than those with the heterozygous (AG) and wide-type genotype (GG). The results could be quite reliable providing that the pooled OR showed statistical significance and the statistical power was nearly 100% under the recessive model. These more refined findings in our study could be useful for future genetic studies on AD.

In order to get more detailed information about this association, we further carried out subgroup analyses by ethnicity and by genotyping method, respectively. On one hand, subgroup analysis by ethnicity indicated that Caucasians might have a lower risk of Alzheimer's disease than Asians (Table 3). In addition, among Caucasians, the British might have much less possibility of suffering from Alzheimer's disease even if their CHAT rs3810950 genotype was AA when compared to the people from other geographic regions (Table 3 and Table S2). Nevertheless, there were only two British studies [20, 21]. This might result in false negative findings and thus people should be more cautious about this result. Low statistical power calculated for the British group clearly supported this point (Table S2). On the other hand, subgroup analysis by genotyping method implied that the genotyping error or bias might exist in our meta-analysis due to the inconsistent results obtained for the quantitative PCR and nonquantitative PCR subgroups, respectively. Besides, the high and low statistical power calculated, respectively, for the quantitative PCR and nonquantitative PCR subgroups could indicate that no association was observed between CHAT rs3810950 polymorphism and AD risk for the nonquantitative PCR subgroup probably just due to its small sample size effect (Table 3).

A further stratified analysis of the association between CHAT rs3810950 allele and Alzheimer's disease according to the ApoE-*ε*4 status showed that, with the same rs3810950 polymorphism, ApoE-*ε*4 carriers exhibited a significantly higher incidence of AD than the non-ApoE-*ε*4 carriers (Table 4). Thus, interaction between CHAT rs3810950 polymorphism and ApoE-*ε*4 allele could be a huge risk factor for Alzheimer's disease. However, only four studies were included in this risk-stratification analysis and we should also treat this result with caution.

Up to date, four independent studies have investigated the link between CHAT rs2177369 polymorphism and the risk of AD. Among them, two studies [21, 22] indicated no relationship between this polymorphism and the susceptibility to the disease. The other two studies [21, 23] found the association between CHAT rs2177369 variant and AD risk but provided the opposite results. Cook et al. [21] denoted that carrying the minor alleles (GG + GA) was significantly protective with respect to carrying the homozygous wide-type allele (AA), while Scacchi et al. [23] showed that the homozygous mutant (GG) was a risk factor compared with the GA + AA genotypes. Our meta-analysis results indicated that the rs2177369 polymorphism played a protective role in the disease, which agreed well with Cook (1) study. Though the results from the overall analysis based on the four independent studies only supported a weak association, the subanalysis of the exclusion of Scacchi study clearly suggested that this polymorphism was a protective factor for AD (Table 5). On the other hand, we considered that the results of no association between CHAT rs2177369 polymorphism and AD risk derived from the subanalysis excluding the Cook (1) study were unreliable since they did not reach the statistical significance and the statistical power was quite low (19.62% for dominant model and 5.97% for recessive model, Table 5). In addition, the substantial heterogeneity existed among the included studies under the allelic, homozygous, and recessive models (Table 5). However, additional studies with larger sample sizes need to be further performed for verifying the potential protective association between CHAT rs2177369 polymorphism and the risk of Alzheimer's disease.

Presence of heterogeneity was detected in this retrospective study. It was known that age, gender, ethnicity, lifestyle habits (smoking and alcohol), education, vascular risk, ApoE-*ε*4 status, and other genetic or environmental factors influence AD onset [31–34]. Therefore, we have reasons to believe that these potential causes may account for the heterogeneity and the different results among the included studies. We tried to extract the data of these AD risk factors for further analysis. However, we could not conduct the subgroup analysis by age, gender, vascular risk, education or habits, and so forth due to their insufficient data. For CHAT rs3810950 polymorphism with AD risk, we could not clarify the sources of significant between-study heterogeneity neither from the ethnicity or genotyping method based subgroup analyses and ApoE-*ε*4-based stratified analysis nor from the meta-regression according to the variables of sample size, ethnicity, and genotyping method. Therefore, other factors such as age, habits, and education may cause the high heterogeneity among studies on rs3810950 polymorphism. For CHAT rs2177369 polymorphism with AD risk, however, we found that substantial heterogeneity only existed between the study investigated by Scacchi et al. [23] and others performed by Cook et al. [21] and Piccardi et al. [22], respectively. When excluding the Scacchi study, only unimportant between-study heterogeneity was observed in the meta-analysis (*I* ^2^ ≤ 26% for all five genetic models, Table 5 and Figure S2). Thus, we also considered that the potential factor such as age, education, habits, or even treatment with anti-inflammatory drugs in the Scacci study, and so forth may account for the high heterogeneity between this study and others on rs2177369 polymorphism.

Our meta-analysis had several advantages. Firstly, this study is the first meta-analysis to investigate the association of CHAT rs2177369 polymorphism with the development of Alzheimer's disease. In addition, though a previous meta-analysis [30] has explored the relationship of CHAT rs3810950 variant with the susceptibility to AD, our study was performed based on a much larger sample size. Secondly, five genetic models were used in this meta-analysis. As a result, our study not only demonstrated the association between these two polymorphisms with AD, but also clarified that the homozygous and heterozygous mutant genotypes might play the potentially different roles in AD susceptibility. Thirdly, subgroup analyses, subanalyses, and meta-regression were conducted to explore the source of heterogeneity under the genetic models in this retrospective analysis. Ethnicity-based subgroup analysis also helped us to investigate the possibly different impact of CHAT rs3810950 polymorphism on AD risk in different ethnic groups. Fourthly, a risk-stratification analysis of the association by ApoE-*ε*4 status was also carried out in our study. This analysis assisted us to detect the promising effect of gene-gene interaction on the development of disease. Fifthly, we not only used Begg's and Egger's tests to assess the risk of publication bias but also employed the trim and fill method to identify and correct funnel plot asymmetry arising from publication bias. Sixthly, the NOS criteria were performed to evaluate the quality of the included studies and Venice criteria were applied to assess the cumulative evidence of the associations in our meta-analysis. Finally, statistical power analysis was also executed to estimate the effect of the sample size on the power of the study and this increased the credibility of our result.

Some limitations should be also recognized in this meta-analysis. Firstly, a subgroup analysis based on age, gender, or lifestyle habits may also contribute to the association of CHAT rs3810950 and rs2177369 polymorphisms with Alzheimer's disease [35, 36], but we did not perform such an extensive analysis because of the limited data. Secondly, the geographic regions of the participants were restricted. We could not find a study on CHAT rs3810950 loci to investigate the population in Africa, Australia, or South America, while studies on CHAT rs2177369 loci are only involved in the British and Italian populations. This limitation might lead our results into less accuracy. Thirdly, we could not use environmental factors to adjust the pooled ORs of the association between the genetic polymorphism and the disease because of the unavailability of environmental information in the included studies. Finally, an obvious heterogeneity was observed in this meta-analysis. The study designs, populations (age and gender), habits, and geographical location may contribute to the heterogeneity.

Alzheimer's disease is the most prevalent neurodegenerative disease in the elderly and it has caused serious damage to our health [1, 2, 6]. Substantial progress has been made towards characterization of Alzheimer's disease, but presently there are still no efficient therapies for Alzheimer's disease, and the pathogenic mechanism of Alzheimer's disease still remains unclear [4, 37, 38]. Thus, it is urgent to enrich our understanding of AD pathogenesis or we will almost surely fail to develop effective treatments for Alzheimer's disease. The results from this meta-analysis would help us to reach this goal.

## Supplementary Material

Considerations for epidemiologic credibility in the assessment of cumulative evidence on genetic associations.

## Figures and Tables

**Table 1:** The baseline characteristics of all the studies included in this meta-analysis.

	First author	Year	Area	Ethnicity	Number of cases	Number of controls	Cases			Controls			HWE	Genotyping	Diagnosis criteria	Statistical power
							G/G	G/A	A/A	G/G	G/A	A/A	*P*	method		Dominant/recessive
rs3810950	Mubumbila [13]	2002	France & Germany	Caucasian	122	112	48	32	42	64	34	14	0.016	SSCP-PCR^1^	None	71.33%/98.68%
	Harold [20]	2003	UK	Caucasian	131	118	69	51	11	65	47	6	0.627	PCR-RFLP^2^	NINCDS-ADRDA	6.62%/22.59%
	Kim [12]	2004	Korea	Asian	246	561	171	61	14	419	133	9	0.856	PCR/sequencing^2^	NINCDS-ADRDA & DSM-IV	33.45%/94.29%
	Cook [21]	2005	UK	Caucasian	210	315	112	76	22	161	128	26	1	PCR-RFLP^2^	NINCDS-ADRDA	8.26%/13.68%
	Ahn Jo [14]	2006	Korea	Asian	316	264	211	99	6	192	70	2	0.129	PCR/sequencing^2^	NINCDS-ADRDA	35.87%/58.33%
	Ozturk [15]	2006	USA	Caucasian	999	708	562	377	60	363	296	49	0.304	PCR/sequencing^2^	NINCDS-ADRDA	51.57%/11.65%
	Tang [16]	2008	China	Asian	273	271	190	75	8	179	83	9	1	PCR-RFLP^2^	NINCDS-ADRDA	14.06%/5.73%
	Grünblatt [17]	2009	Austria	Caucasian	120	456	63	45	12	268	164	24	1	TaqMan^1^	NINCDS-ADRDA	23.56%/47.82%
	Grünblatt [18]	2011	Austria & Italy	Caucasian	137	464	79	46	12	267	173	24	0.61	TaqMan^1^	NINCDS-ADRDA	5.00%/35.07%
	Mengel-From [10]	2011	Denmark	Caucasian	661	1308	38	203	420	55	455	798	0.369	TaqMan^1^	NINCDS-ADRDA	90.29%/7.57%
	Lee [19]	2012	Korea	Asian	736	1386	505	205	26	1023	342	21	0.26	RT-PCR^1^	None	73.11%/94.39%

rs2177369	Cook (1) [21]	2005	UK	Caucasian	202	295	29	85	88	76	124	95	0.0073	PCR-RFLP^2^	NINCDS-ADRDA	77.81%/71.82%
	Cook (2) [21]	2005	UK (Cardiff)	Caucasian	179	175	26	79	74	29	83	63	0.8754	PCR-RFLP^2^	NINCDS-ADRDA	17.71%/7.82%
	Piccardi [22]	2007	Italy	Caucasian	158	118	44	75	39	40	57	21	1	PCR-RFLP^2^	NINCDS-ADRDA & DSM-IV	36.06%/15.30%
	Scacchi [23]	2009	Italy	Caucasian	442	218	167	200	75	61	117	40	0.2737	PCR-RFLP^2^	NINCDS-ADRDA	8.45%/56.24%

Note: ^1^quantitative PCR and ^2^nonquantitative PCR.

**Table 2:** Quality assessment scheme for the included literatures (Newcastle-Ottawa Scale).

First author	Year	Selection				Comparability	Exposure			Total
		I	II	III	IV	V	VI	VII	VIII	
Mubumbila [13]	2002	*∗*	*∗*		*∗*	*∗*		*∗*		*∗∗∗∗∗*
Harold [20]	2003	*∗*	*∗*		*∗*	*∗*		*∗*		*∗∗∗∗∗*
Kim [12]	2004	*∗*	*∗*	*∗*	*∗*	*∗∗*		*∗*		*∗∗∗∗∗∗∗*
Cook [21]	2005	*∗*	*∗*		*∗*	*∗*		*∗*		*∗∗∗∗∗*
Ahn Jo [14]	2006	*∗*	*∗*	*∗*	*∗*	*∗∗*		*∗*		*∗∗∗∗∗∗∗*
Ozturk [15]	2006	*∗*	*∗*		*∗*	*∗∗*		*∗*		*∗∗∗∗∗∗*
Tang [16]	2008	*∗*	*∗*		*∗*	*∗∗*		*∗*		*∗∗∗∗∗∗*
Grünblatt [17]	2009	*∗*	*∗*		*∗*	*∗*		*∗*		*∗∗∗∗∗*
Grünblatt [18]	2011	*∗*	*∗*		*∗*	*∗*		*∗*		*∗∗∗∗∗*
Mengel-From [10]	2011	*∗*	*∗*		*∗*	*∗∗*		*∗*		*∗∗∗∗∗∗*
Lee [19]	2012	*∗*	*∗*	*∗*	*∗*	*∗∗*		*∗*	*∗*	*∗∗∗∗∗∗∗∗∗*
Piccardi [22]	2007	*∗*	*∗*		*∗*	*∗*		*∗*		*∗∗∗∗∗*
Scacchi [23]	2009	*∗*	*∗*		*∗*	*∗*		*∗*		*∗∗∗∗∗*

Note: I: is the case definition adequate? II: representativeness of the cases. III: selection of controls. IV: definition of controls. V: comparability of cases and controls on the basis of the design or analysis. VI: ascertainment of exposure. VII: same method of ascertainment for cases and controls. VIII: nonresponse rate.

**Table 3:** Subgroup analyses of the association between CHAT rs3810950 polymorphism and Alzheimer's disease risk.

	Genetic comparison	*I* ^2^ (%)	Effect model	OR [95% CI]	*P*_OR_	Statistical power
Overall	A versus G	74	Random	1.18 [1.01, 1.37]	0.03	NA
	AA versus GG	72	Random	1.63 [1.09, 2.42]	0.02	NA
	AG versus GG	37	Fixed	0.99 [0.90, 1.10]	0.87	NA
	AA + GA versus GG	62	Random	1.08 [0.92, 1.28]	0.34	46.23%
	AA versus GG + GA	66	Random	1.65 [1.20, 2.26]	<0.01	99.99%

Ethnicity-based						
Caucasian (7)	A versus G	77	Random	1.16 [0.94, 1.42]	0.16	NA
	AA versus GG	74	Random	1.42 [0.90, 2.25]	0.13	NA
	AG versus GG	0	Fixed	0.88 [0.77, 1.00]	0.06	NA
	AA + GA versus GG	60	Random	1.02 [0.82, 1.28]	0.85	6.34%
	AA versus GG + GA	66	Random	1.47 [1.05, 2.07]	0.03	99.99%

Ethnicity-based						
Asian (4)	A versus G	52	Random	1.23 [1.01, 1.48]	0.04	NA
	AA versus GG	46	Fixed	2.24 [1.48, 3.39]	<0.01	NA
	AG versus GG	5	Fixed	1.15 [0.99, 1.32]	0.07	NA
	AA + GA versus GG	33	Fixed	1.21 [1.06, 1.40]	<0.01	77.90%
	AA versus GG + GA	40	Fixed	2.18 [1.45, 3.29]	<0.01	99.45%

Genotyping-based						
Quantitative PCR (5)	A versus G	76.5	Random	1.32 [1.05, 1.65]	0.02	NA
	AA versus GG	80.6	Random	1.89 [1.00, 3.55]	0.05	NA
	AG versus GG	46.9	Fixed	1.08 [0.93, 1.26]	0.32	NA
	AA + GA versus GG	64.3	Random	1.18 [0.90, 1.55]	0.24	77.24%
	AA versus GG + GA	77.3	Random	1.94 [1.18, 3.19]	0.01	99.99%

Genotyping-based						
Nonquantitative PCR (6)	A versus G	62.8	Random	1.06 [0.88, 1.28]	0.52	NA
	AA versus GG	61.6	Random	1.40 [0.82, 2.37]	0.22	NA
	AG versus GG	18.8	Fixed	0.93 [0.82, 1.06]	0.29	NA
	AA + GA versus GG	48.4	Fixed	1.01 [0.83, 1.21]	0.95	5.11%
	AA versus GG + GA	55.7	Random	1.42 [0.88, 2.29]	0.16	75.03%

Note: NA: not applicable.

**Table 4:** Risk of Alzheimer's disease associated with CHAT rs3810950 polymorphism by ApoE-*ε*4 status.

Genetic comparison	Non-ApoE-*ε*4 carriers				ApoE-*ε*4 carriers			
	Cases	Controls	OR (95% CI)	*P*	Cases	Controls	OR (95% CI)	*P*
GG + GA	851	1605	1 (reference)	NA	862	377	3.46 (1.78–6.71)	<0.001
AA	292	673	1.03 (0.62–1.71)	0.08	203	185	4.87 (1.67–14.22)	0.004

Note: NA: not applicable.

**Table 5:** Meta-analysis of the association between CHAT rs2177369 polymorphism and Alzheimer's disease risk.

Meta-analysis	Genetic comparison	*I* ^2^ (%)	Effect model	OR [95% CI]	*P*_OR_	Statistical power
Overall	G versus A	82	Random	0.85 [0.61, 1.18]	0.34	NA
	GG versus AA	77	Random	0.73 [0.40, 1.32]	0.3	NA
	GA versus AA	0	Fixed	0.80 [0.63, 1.01]	0.06	NA
	GG + GA versus AA	32	Fixed	0.77 [0.62, 0.96]	0.02	69.05%
	GG versus AA + GA	82	Random	0.85 [0.49, 1.47]	0.55	30.29%

Analysis without Cook (1) study	G versus A	72	Random	0.96 [0.70, 1.31]	0.78	NA
	GG versus AA	62	Random	0.91 [0.52, 1.59]	0.73	NA
	GA versus AA	0	Fixed	0.83 [0.62, 1.10]	0.19	NA
	GG + GA versus AA	7	Fixed	0.87 [0.67, 1.14]	0.32	19.62%
	GG versus AA + GA	69	Random	1.04 [0.63, 1.71]	0.88	5.97%

Analysis without Scacchi study	G versus A	22	Fixed	0.73 [0.61, 0.86]	<0.01	NA
	GG versus AA	13	Fixed	0.54 [0.39, 0.76]	<0.01	NA
	GA versus AA	0	Fixed	0.76 [0.58, 0.99]	0.05	NA
	GG + GA versus AA	0	Fixed	0.68 [0.53, 0.88]	<0.01	86.83%
	GG versus AA + GA	26	Fixed	0.65 [0.48, 0.87]	<0.01	73.79%

Note: NA: not applicable.
