# Supplementary material for: Genetic Association of CHAT rs3810950 and rs2177369 Polymorphisms with the Risk of Alzheimer's Disease: A Meta-Analysis
Source: Biomed Res Int. 2016 Aug 15;2016:9418163. doi: 10.1155/2016/9418163 (PMC5002460; doi:10.1155/2016/9418163)
Supplement: Supplementary file 1 — Considerations for epidemiologic credibility in the assessment of cumulative evidence on genetic associations. [file 9418163.f1.pdf]

"Supporting Information"

Genetic association of CHAT rs3810950 and rs2177369 polymorphisms with the risk of Alzheimer's disease: a meta-analysis

Yong Liu[&], Qicong Chen[&], Xu Liu, Mengmeng Dou, Silu Li, Jiahui Zhou, Hong Liu, Yongfu Wu[*], Zunnan Huang*

[&] Equal contributors

***Corresponding Authors**: Zunnan Huang, Yongfu Wu

Table S1. Considerations for epidemiologic credibility in the assessment of cumulative evidence on genetic associations[1].

Criteria	Categories	Proposed operationalization
Amount of evidence	A: Large-scale evidence<br>B: Moderate amount of evidence<br>C: Little evidence	Thresholds may be defined based on sample size, power or false-discovery rate considerations. The frequency of the genetic variant of interest should be accounted for. As a simple rule, we suggest that category A requires over 1000 subjects (total number of cases and controls assuming 1:1 ratio) evaluated in the least common genetic group of interest; B corresponds to 100–1000 subjects evaluated in this group and C corresponds to <100 subjects evaluated in this group (see 'Discussion' section in the text and Table 2 for further elaboration).[a]
Replication	A: Extensive replication including at least one well-conducted meta-analysis with little between-study inconsistency<br>B: Well-conducted meta-analysis with some methodological limitations or moderate between-study inconsistency<br>C: No association; no independent replication; failed replication; scattered studies; flawed meta-analysis or large inconsistency	Between-study inconsistency entails statistical considerations (e.g. defined by metrics such as I^2, where values of 50% and above are considered large and values of 25–50% are considered moderate inconsistency) and also epidemiological considerations for the similarity/standardization or at least harmonization of phenotyping, genotyping and analytical models across studies.<br>See 'Discussion' section in the text for the threshold (statistical or others) required for claiming replication under different circumstances (e.g. with or without including the discovery data in situations with massive testing of polymorphisms).
Protection from bias	A: Bias, if at all present, could affect the magnitude but probably not the presence of the association<br>B: No obvious bias that may affect the presence of the association but there is considerable missing information on the generation of evidence<br>C: Considerable potential for or demonstrable bias that can affect even the presence or absence of the association	A prerequisite for A is that the bias due to phenotype measurement, genotype measurement, confounding (population stratification) and selective reporting (for meta-analyses) can be appraised as not being high (as shown in detail in Table 3) plus there is no other demonstrable bias in any other aspect of the design, analysis or accumulation of the evidence that could invalidate the presence of the proposed association. In category B, although no strong biases are visible, there is no such assurance that major sources of bias have been minimized or accounted for because information is missing on how phenotyping, genotyping and confounding have been handled. Given that occult bias can never be ruled out completely, note that even in category A, we use the qualifier 'probably'.

[a]For example, if the association pertains to the presence of homozygosity for a common variant and if the frequency of homozygosity is 3%, then category A amount of evidence requires over 30 000 subjects and category B between 3000 and 30 000.

Table S2. Meta-analysis of the association between CHAT rs3810950 polymorphism and AD risk in the British population.

Genetic comparison	I^2 (%)	Effect Model	OR[95%CI]	P_{OR}	Statistical power
A *vs.* G	0	Fixed	1.05 [0.84, 1.31]	0.68	
AA *vs.* GG	0	Fixed	1.33 [0.78, 2.27]	0.29	
AG *vs.* GG	0	Fixed	0.91 [0.67, 1.23]	0.53	
AA+GA *vs.* GG	0	Fixed	0.97 [0.73, 1.30]	0.85	5.41%
AA *vs.* GG+GA	0	Fixed	1.39 [0.83, 2.34]	0.21	26.56%

Table S3. Assess the credibility of the cumulative evidence of the meta-analyzed associations according to Venice criteria.

Genetic comparisons	A *vs.* G			AA *vs.* GG			AG *vs.* GG			AA+AG *vs.* GG			AA *vs.* AG+GG		
Grade	AE	CR	PB	AE	CR	PB	AE	CR	PB	AE	CR	PB	AE	CR	PB
Overall analysis	A	C	A	A	C	A	A	B	A	A	C	A	A	C	A
Subgroup analyses															
Asian	A	C	NA	A	B	NA	A	B	NA	A	B	NA	A	A	NA
Caucasian	A	C	NA	A	C	NA	A	B	NA	A	C	NA	A	C	NA
Quantitative PCR	A	B	NA	A	A	NA	A	A	NA	A	A	NA	A	A	NA
Non-quantitative PC	A	A	NA	A	A	NA	A	C	NA	A	A	NA	A	A	NA
British	B	A	NA	B	A	NA	B	A	NA	B	A	NA	B	A	NA

Note: AE: amount of evidence; CR: consistency of replication;
PB: protection from bias; NA: not applicable.

Table S4. Reported results of Begg's test and Egger's test to assess the publication bias of the include studies under the genetic models of the association between CHAT rs3810950 polymorphism and AD risk.

Genetic comparison	Begg's test(z, p)	Egger's test(t, p）
A *vs.* G	1.25, 0.213	1.71, 0.122
AA *vs.* GG	0.93, 0.350	2.26, 0.050
AG *vs.* GG	0.00, 1.000	-0.06, 0.954
AA+GA *vs.* GG	0.31, 0.755	0.65, 0.535
AA *vs.* GG+GA	0.78, 0.436	2.34, 0.044

Figure S1. Forest plots of analysis without Cook(1) study between CHAT rs2177369 polymorphism and AD risk in five genetic models. **A:** the allelic model (G *vs.* A); **B:** the homozygous model (GG *vs.* AA); **C:** the heterozygous model (GA *vs.* AA); **D:** the dominant model (GG+GA *vs.* AA); **E:** the recessive model (GG *vs.* GA+AA).

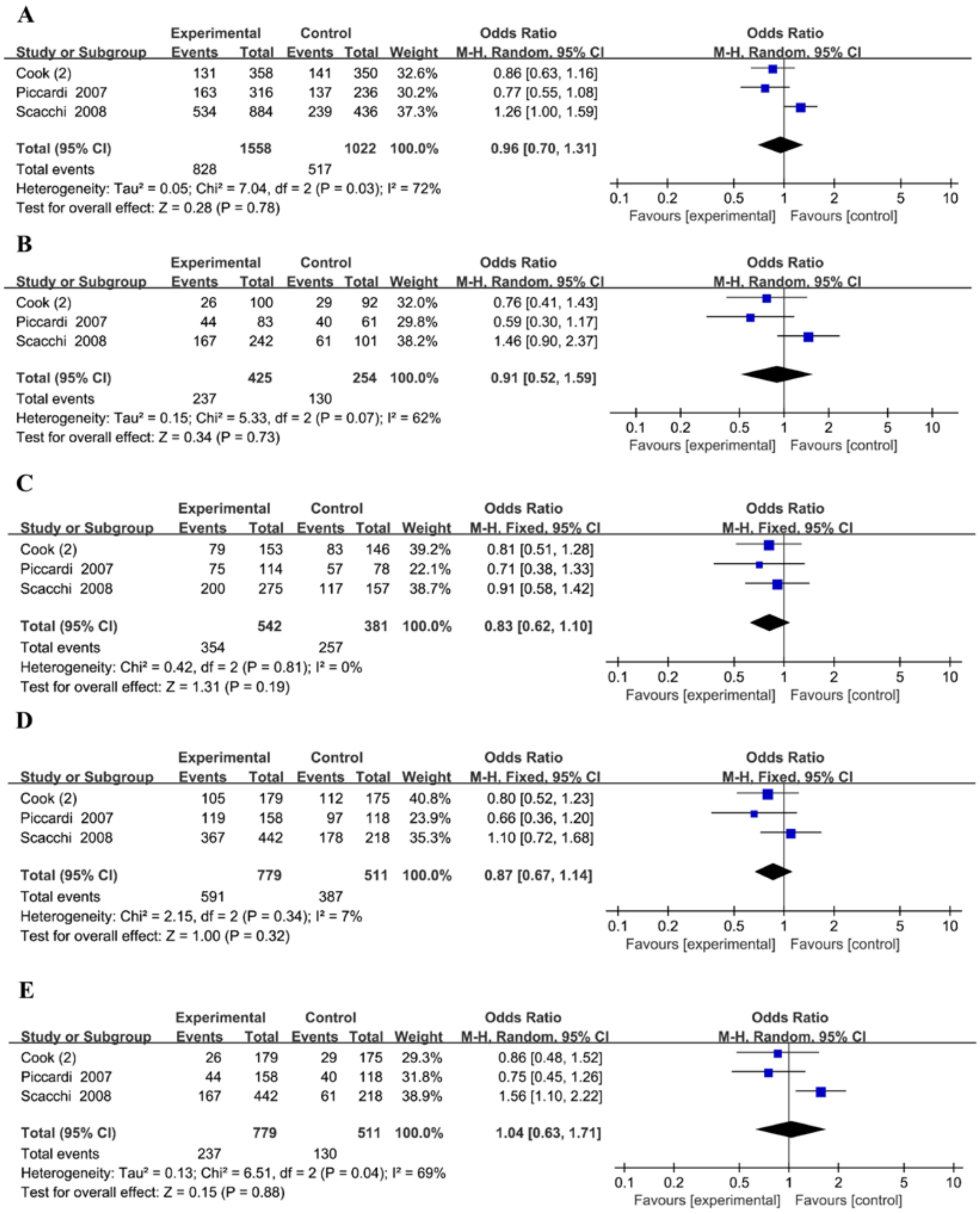

Figure S2. Forest plots of analysis without Scacchi study between CHAT rs2177369 polymorphism and AD risk in five genetic models. **A:** the allelic model (G *vs.* A); **B:** the homozygous model (GG *vs.* AA); **C:** the heterozygous model (GA *vs.* AA); **D:** the dominant model (GG+GA *vs.* AA); **E:** the recessive model (GG *vs.* GA+AA).

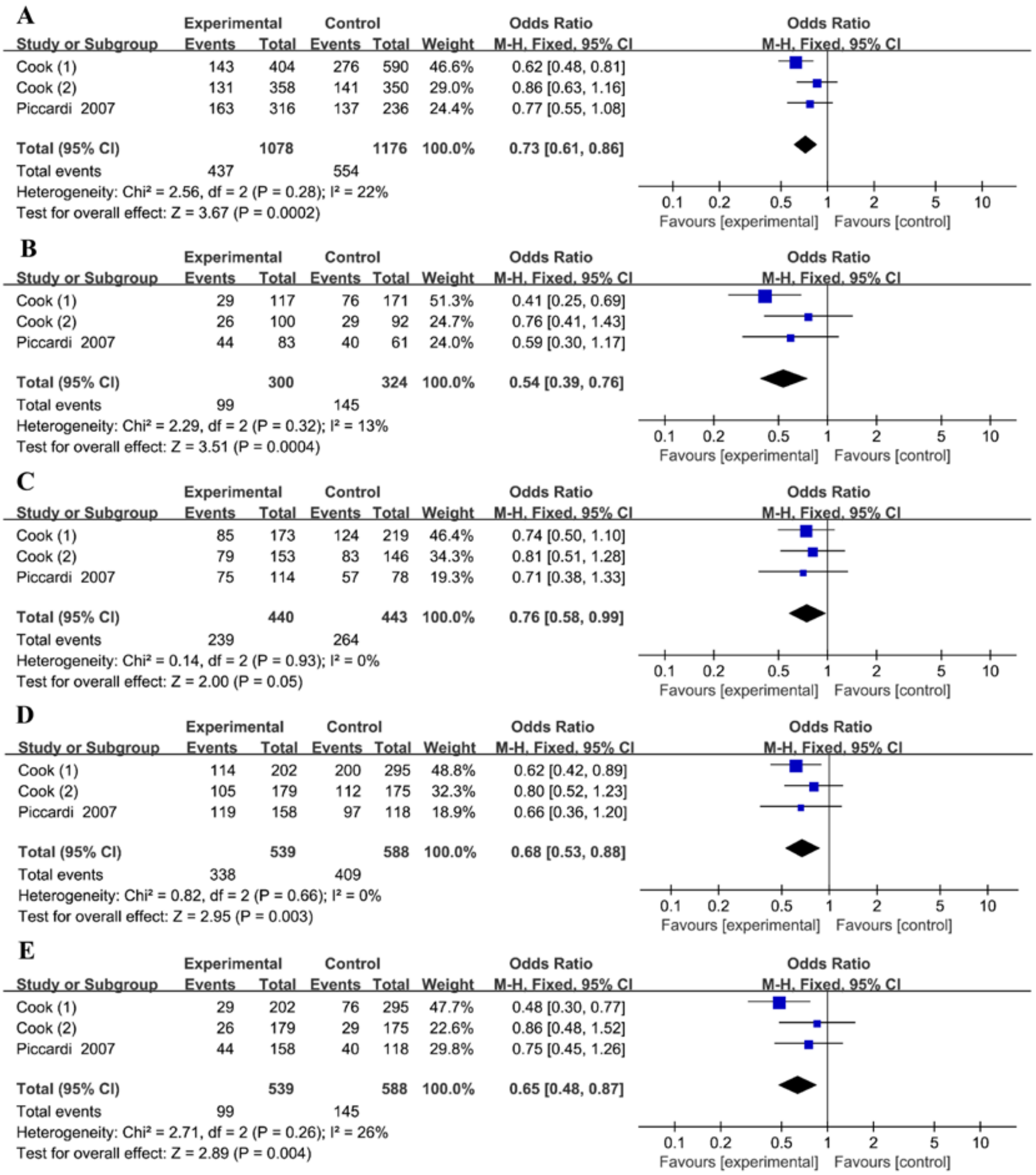

Fig S3. Funnel plot of CHAT rs3810950 polymorphism and AD risk in the heterozygote model (AG *vs.* GG).

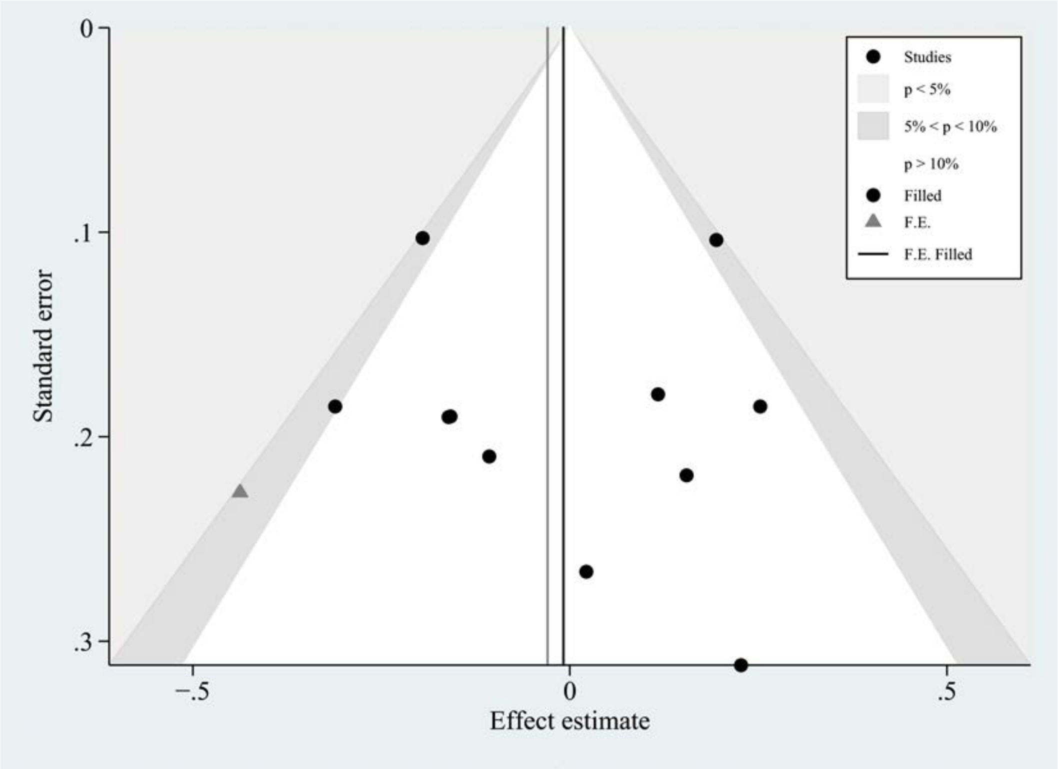

1. Ioannidis, J.P., et al., *Assessment of cumulative evidence on genetic associations: interim guidelines.* International journal of epidemiology, 2008. **37**(1): p. 120-132.
